# µRT: A lightweight real-time middleware with integrated validation of timing constraints

**DOI:** 10.3389/frobt.2023.1081875

**Published:** 2023-03-21

**Authors:** Thomas Schöpping, Svenja Kenneweg, Marc Hesse, Ulrich Rückert

**Affiliations:** Cognitronics and Sensor Systems Group, Faculty of Technology, Bielefeld University, Bielefeld, Germany

**Keywords:** middleware, real-time computing, distributed computing, embedded, microcontroller, publish–subscribe, remote procedure call, prevention through design

## Abstract

Middlewares are standard tools for modern software development in many areas, especially in robotics. Although such have become common for high-level applications, there is little support for real-time systems and low-level control. Therefore, µRT provides a lightweight solution for resource-constrained embedded systems, such as microcontrollers. It features publish–subscribe communication and remote procedure calls (RPCs) and can validate timing constraints at runtime. In contrast to other middlewares, µRT does not rely on specific transports for communication but can be used with any technology. Empirical results prove the small memory footprint, consistent temporal behavior, and predominantly linear scaling. The usability of µRT was found to be competitive with state-of-the-art solutions by means of a study.

## 1 Introduction

For sophisticated software architectures, middlewares have become important tools to facilitate modular systems, which—despite their complexity—are easy to maintain and can be extended with minimal effort. Although numerous solutions have been developed in the last decades, only a few consider real-time computing. Regarding robotic systems, which interact with their environment on a physical level, this paradigm of computer science is inevitable, though, as it is a vital requirement for safe operation. In contrast to other domains that require real-time processing, modern robotic platforms need high modularity and complete determinism regarding execution and reaction times to be adaptable and extensible but also safe (Stankovic, 1988; Shin and Ramanathan, 1994; Oshana, 2006; Wörn, 2006; Zurawski, 2006; Jahn, 2021).

The novel middleware presented in this work has its roots in just this challenge to combine both worlds in a single system. When working with the AMiRo platform (Herbrechtsmeier et al., 2016; Herbrechtsmeier, 2017), which features a heterogeneous, distributed real-time architecture, the application development became disproportionally difficult as complexity increased. The monolithic software design did not resemble the modular hardware, nor could it satisfy fundamental use cases for the robot. AMiRo features multiple microcontrollers (MCUs), which form a loosely coupled real-time system, but each of which is responsible for multiple tasks, such as power management, motor control, sensor fusion, wireless communication, and behavioral applications (Schöpping et al., 2015; Schöpping et al., 2018; Korthals et al., 2019; Schöpping and Kenneweg, 2022a; *cf.* Figure 12). By introducing a communication middleware to the real-time level of the platform, these issues should be resolved, and several existing solutions have been evaluated. Unfortunately, none could satisfy all requirements, which eventually led to the decision to develop a completely new system: µRT (pronounced like “Marty”: [má:rti]).

Before µRT is described in detail in section 2, several types of existing middlewares and fundamental concepts are presented in this section (*cf.* sections 1.1 and 1.2). In section 3, µRT is evaluated thoroughly in three aspects: feature set (*cf.* section 3.1), performance (*cf.* section 3.2), and usability for software developers (*cf.* section 3.3). The findings are briefly discussed in section 4 before conclusions about µRT are drawn in section 4.1, and future enhancements are proposed in section 4.2.

### 1.1 Related work

Today, it is very common to use middlewares for communication in modular architectures. During the last decades, a great number of middlewares have been developed, with CORBA (Yang and Duddy, 1996), MQTT (Stanford-Clark and Hunkeler, 1999), and ROS (Quigley et al., 2009; Macenski et al., 2022) being some of the most popular ones. Using such tools has numerous advantages:

• Compatible applications can be executed on any system that runs the according middleware, allowing for high code portability.
• Existing software can be reused and integrated with minimal effort.
• Realization of further applications is simplified due to uniform interfaces and additional debugging and profiling tools most solutions provide, leading to high-quality code while minimizing development time.

One major issue with existing middlewares is that only very few consider real-time computing. Thus, most cannot be used for such use cases. There are exceptions to this rule, such as real-time CORBA (Fay-Wolfe et al., 2000), Orocos (Bruyninckx, 2001), R2P (Migliavacca, 2013; Matteucci et al., 2015; Migliavacca, 2016), and ROS 2 (Macenski et al., 2022), although those have other disadvantages. Notably, neither of the aforementioned solutions features actual validation of timing constraints at runtime. However, for large, potentially harmful, or even lethal platforms, the detection of real-time violations is a crucial requirement for safe operation. Even though static scheduling techniques exist to determine a valid task execution sequence and prevent timing violations at runtime, methods and solutions become much more complex for distributed systems and require a high level of control over individual components (Di Natale and Stankovic, 2000; Zurawski, 2006).

#### 1.1.1 Real-time CORBA

Although CORBA is actually an open standard defined by the Object Management Group (OMG, 2021), it contains several design flaws, which are consequently inherited by all implementations, such as TAO (Schmidt et al., 1998), TAOX11 (Remedy IT, 2019), or omniORB (Grisby, 2022). Due to its design by committee, it suffers from several issues regarding complexity, redundancy, and missing features (Henning, 2006). Because real-time CORBA is a modification of the original specification (OMG, 2005), it incorporates the same issues, rendering it a suboptimal solution. Especially when targeting resource-constrained platforms such as MCUs, the high complexity of CORBA inevitably results in high resource requirements, which such devices can rarely (and if so, just barely) satisfy. As a result, all implementations of newer versions of the CORBA specifications only support sophisticated operating systems, such as Linux or Windows, but are not designed to be deployed on MCUs.

#### 1.1.2 Orocos

Originally developed by Bruyninckx (2001), the Orocos project is a collection of libraries and tools for the efficient development of robotics software, which is portable and has high runtime performance, with real-time support being one of its core aspects. Moreover, it can be combined with other middlewares if desired. For certain communication schemes, Orocos employs CORBA (*cf.* section 1.1.1; optional for local, non-distributed setups), and it can also be integrated with ROS (Quigley et al., 2009) and ROS 2 (Macenski et al., 2022). However, Orocos is not designed to be deployed on MCUs and does not feature validation of timing constraints at runtime.

#### 1.1.3 R2P

R2P has been developed by Migliavacca (2013) as an alternative to ROS (Quigley et al., 2009), LCM (Huang et al., 2010), and FAMOUSO (Schulze and Zug, 2008; Schulze, 2009) with improved support for hard real-time systems. It is focused to be used on MCUs, which communicate *via* a controller area network (CAN), specifically the RTCAN protocol (Migliavacca et al., 2013). As such, it seemed appropriate for the AMiRo platform at first glance, as it is also based on ChibiOS (di Sirio, 2020, 2022), just like AMiRo-OS (Schöpping et al., 2016), the real-time operating system (RTOS) of AMiRo. Unfortunately, R2P relies on dynamic memory allocation as it employs memory pools. Therefore, complete determinism cannot be guaranteed. Because the development of the project ceased in 2016, there was no support to implement the according modifications, and there is little detailed documentation about the project to be found.

#### 1.1.4 ROS 2

The arguably most popular solution in robotics is ROS, which introduced real-time support with ROS 2 (Quigley, 2015; Macenski et al., 2022) and its extensions, micro-ROS (OSRF, 2022a) and RT-ROS (Wei et al., 2016; Faust, 2022), respectively. However, the overall performance of ROS 2 strongly depends on the utilized data distribution service (DDS; OMG, 2015) implementation (Maruyama et al., 2016). Regarding MCUs and micro-ROS, DDS for eXtremely Resource Constrained Environments (DDS-XRCE; OMG, 2019) must be employed, which specifies a centralized communication topology with multiple clients interacting with a single server—an undesired architecture for distributed systems composed of equal participants. Because its popularity makes it the *de facto* standard solution in robotics, ROS 2 has been evaluated to a particular extent.

**FIGURE 1:**
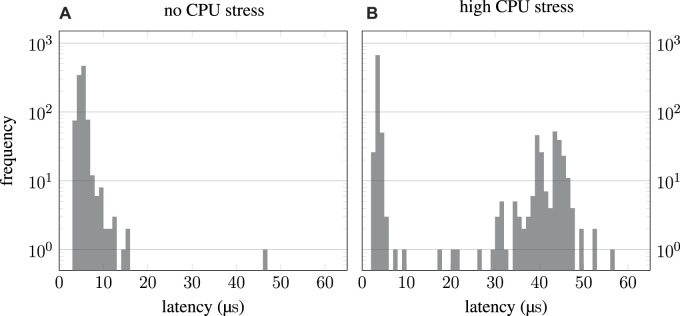
ROS 2 “pendulum demo” benchmark results. While the system^1^ was only running the “pendulum demo” for the left histogram **(A)**, additional stress was put on the CPU for the right-hand results **(B)**. Although the results presented by Kay and Tsouroukdissian (2015) are reproduced very well, logarithmic scaling of the frequency axis reveals a significant number of samples with high latency.

Although the ROS ecosystem is generally very powerful, there are indications that the real-time capabilities of ROS 2 are still not optimal. First, Kay and Tsouroukdissian (2015) presented empirical results of the official ROS 2 demo application for real-time use cases “pendulum demo,” which were promising overall. However, they showed occasional latency spikes, especially when the CPU was put under load. Because these benchmarks are rather dated and real-time capabilities might have been optimized since, another set of benchmarks was conducted,^1^ which still confirms the limited suitability of ROS 2 for hard real-time applications. The results of these benchmarks are depicted in Figure 1 and reveal two important findings. The histograms show the results of the same benchmarks as presented by Kay and Tsouroukdissian (2015), and the original result data is resembled very well. However, no “outliers” were visible in the presentation due to the linear scaling of the frequency axis. When the CPU is put under load, these “outliers” become even more frequent and resemble a Gaussian distribution. This is problematic regarding real-time systems because latency is not limited by an upper bound. The issue becomes even more evident when considering the goal of a jitter of fewer than 30 µs (3% of 1 ms period), as defined by Kay and Tsouroukdissian (2015). For the benchmark with additional CPU load, almost 25% of all data points violate that constraint. Although the cause for the worse performance, despite process priorities configured in favor of the “pendulum demo” (real-time vs. nice), may be rooted in the OS rather than ROS 2, there are obviously no mechanisms in place to limit execution times or at least notify about high latencies.

Further evidence of the limited real-time capabilities of ROS 2 can be found in the performance analyses by Maruyama et al. (2016). The data reveal that ROS 2 scales worse than linear in many situations. Hence, strict timing constraints become exponentially more difficult to meet when system complexity increases. The data also show strong latency variations, even exceeding 10% of the median for data sizes of 1 MB and more. Overall, the real-time capabilities of ROS 2 remain insufficient for scenarios where timing constraints are critical and must be respected and its high resource requirements render it unsuitable for MCUs without sacrificing decentralization.

### 1.2 Interaction concepts

When designing a new midleware, several alternate paradigms can be followed. Eugster et al. (2003) gave a comprehensive overview, and the most important concepts are summarized as follows.

#### 1.2.1 Decoupling

One of the most important characteristics of any distributed communication framework is decoupling. Participants can be coupled in three domains, all of which should be omitted:

• space: If participants must know each other in order to exchange information, they are coupled in space.
• time: In case producers and consumers both have to be active and connected when data is transmitted, they are coupled in time.
• synchronization: For systems coupled in this domain, execution is blocked when sending or receiving data.

#### 1.2.2 Interaction

Furthermore, Eugster et al. (2003) described six fundamental concepts of interaction and how communication between participants is realized. The publish–subscribe paradigm eventually achieves decoupling in all three domains and thus is the most powerful technique in this regard. Another very popular concept is remote procedure calls (RPCs), tightly coupled according to Eugster et al. (2003), but allowing for “pulling” communication, whereas information can only be “pushed” with publish–subscribe. Fortunately, the coupling can be relaxed by an extension of RPC, called “future” (Ananda et al., 1992) or “wait-by-necessity” (Caromel, 1993; *cf.* Eugster et al., 2003).

#### 1.2.3 Addressing

In order to establish communication between producers and consumers in a decoupled manner, addressing information needs to be abstracted. There are several approaches to how this can be realized (Eugster et al., 2003).

• topic/service-based: Producers provide information *via* a certain topic or service, usually identified by a name. Consumers can declare interest in specific information by such identifiers and will eventually receive all data provided *via* the according topic/service.
• content-based: Consumers can define a set of rules (or filters) on whether they will receive new data. Only if the content of a message meets these rules, it (the message) is delivered to the consumer.
• type-based: When a producer emits a message with a complex data type as payload, a consumer might be interested but in a subset of that data. Hence, only this part of the original message is delivered to that specific consumer.

Hybrid techniques are also possible, such as the scope-based approach of Robotics Service Bus (RSB) (Wienke and Wrede, 2011). It employs a URI format for scope names, introducing a hierarchy and filtering capabilities without defining any requirements on the actual information payload.

#### 1.2.4 Quality of Service

Quality of service (QoS) is typically used to track information such as latencies and the number of delivered and discarded messages*.* However, timing constraints are of major importance to guarantee system stability and safety regarding real-time systems. Therefore, the according mechanisms can prioritize important communication and preempt others. Furthermore, the system can be monitored to detect critical failures (i.e., timing violations) as soon as possible and initiate an appropriate reaction. Hence, when timing constraints are not met, temporal behavior tracking and execution of defined routines are also part of QoS in the nomenclature of this work.

## 2 µRT

Although the original motivation for the development of a new middleware was the modularization of the software running on the MCUs of AMiRo (Herbrechtsmeier et al., 2016; Herbrechtsmeier, 2017), several additional goals were specified to make the resulting system applicable for a wide range of other devices with very strict real-time requirements:

1. Memory footprint small enough for mainstream and ultra-low-power MCUs.
2. Throughout real-time capability per completely deterministic and very consistent behavior at runtime.
3. At most linear scaling with increasing system complexity (e.g., number of participants).
4. Validation of timing constraints at runtime.
5. Support of periodic/time-based and event-based communication schemes.
6. Easy-to-use interfaces that help developers create correct and efficient code.
7. Interoperability with existing middleware.
8. High configurability to adapt the system to any specific use case.

**FIGURE 2:**
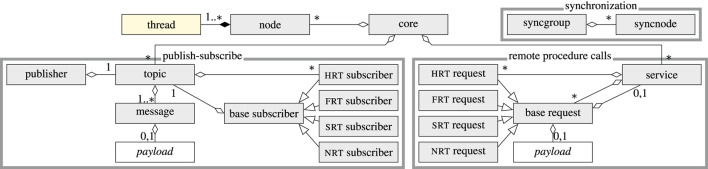
µRT architecture overview. While the *thread* type is defined by the underlying operating system *via* OSAL and *payload* are individual types for each *topic* and *request*, all other components are specified by µRT.

The proposed solution to that challenge—µRT—is an entirely event-based system, featuring a low memory footprint, full real-time capabilities, and built-in validation of timing constraints at runtime. It features a topic-based publish–subscribe architecture and future RPCs (*cf.* section 1.2). It is implemented^2^ in C (Schöpping and Kenneweg, 2022a) and declares all interface functions to external components by its operating system abstraction layer (OSAL), as described in section 2.4. The implementation is highly configurable at compile time through a comprehensive set of feature flags, which allow disabling entire subsystems (e.g., publish–subscribe or RPC) to reduce memory footprint and improve performance. An overview of the µRT architecture is depicted in Figure 2. Before the several components are described in detail in sections 2.5–2.7, some basic concepts about its real-time classes and constraints and the fundamental approach of µRT are presented in sections 2.1–2.3. Finally, the interface-agnostic approach to interacting with other components (e.g., foreign middlewares) in sophisticated, complex systems is described in section 2.8.

### 2.1 Types of constraints

µRT defines three types of timing constraints: latency, synchronicity, and rate. If a constraint is met, its validation function results to 1, whereas a value of 0 indicates a timing violation.

#### 2.1.1 Latency

Often referred to as “deadline,” a maximum expected latency *τ* for information propagation can be specified. It is defined as a function *l*(*τ*, Δ*t*) with Δ*t* the duration of an operation so far:

$$l\left(\tau ,\Delta t\right)=\left\{\begin{array}{cc} 1,\; & \text{if }\Delta t\leq \tau \\ 0,\; & \text{otherwise} \end{array}\right.$$ (1)

#### 2.1.2 Synchronicity

For periodic tasks, it is often required that all iterations take similarly long and execution time varies as little as possible. For real-time systems, this “jitter” must be limited by an upper bound *δ*. By tracking the minimum and maximum iteration times (*t*_min_ and *t*_max_) during system operation, the validity of the synchronicity constraint *s*(*δ*, *t*_min_, *t*_max_) can be calculated by

$$s\left(\delta ,t_{\text{min}},t_{\text{max}}\right)=\left\{\begin{array}{cc} 1,\; & \text{if }t_{\text{max}}-t_{\text{min}}\leq \delta \\ 0,\; & \text{otherwise} \end{array}\right.$$ (2)

#### 2.1.3 Rate

Although this constraint is not considered at all by most authors, it is crucial to detect the complete failure of individual components in an event-driven system at runtime. If a data source does not provide any further information, no data processing pipeline will be triggered, and neither latency nor synchronicity constraints will ever be violated. A possible solution to this challenge is the implementation of a dead man’s switch, which is monitored and checked for regular activation by another component. Alternatively, rate constraints can be validated without the need for a dedicated monitor by introducing a maximum period *ϵ* between subsequent data points and comparison of the current time *t* and the time of the latest data point *t*_*i*−1_:

$$r\left(\epsilon ,t_{i-1},t\right)=\left\{\begin{array}{cc} 1,\; & \text{if }t-t_{i-1}\leq \epsilon \\ 0,\; & \text{otherwise} \end{array}\right.$$ (3)

### 2.2 Real-time classes

µRT distinguishes four classes of real time: hard, firm, soft, and non-real-time. Most authors consider only two classes—hard and soft—because the other definitions are special cases of the latter (Oshana, 2006; Wörn, 2006). However, from an implementation point of view, it makes sense to consider all four cases. The common ground for all classes is that usefulness *u* ∈ [0, 1] is calculated so that the major differences are further restrictions in the mapping functions and interpretation of *u*.

#### 2.2.1 Non-real-time (NRT)

This trivial class has no real-time constraints at all. As a result, *u* = 1 always holds.

#### 2.2.2 Soft real-time (SRT)

As the most general class, the mapping Δ*t*↦*u* can be of any form for SRT. Each component may define an individual mapping function to calculate *u* in order to assess the quality of each data point during operation. While monotonic decreasing functions are most common, partial functions can be used to model desired temporal frames (i.e., to represent jitter constraints). Most notably, the NRT and FRT classes are, in fact, special cases of SRT.

#### 2.2.3 Firm real-time (FRT)

This class further restricts *u* to be either 1 or 0 (“valid” or “invalid”). Such a distinction makes sense from an implementation perspective for two reasons. Calculations and representations do not require “sophisticated” data types, such as float. Because many MCUs do not feature an FPU (floating-point unit), emulation of such types is computationally expensive and may result in temporal inconsistency. Furthermore, this allows for a general calculation of *u*, depending on constraints for latency, synchronicity, and rate:

$$u=l\left(\tau ,\Delta t\right)\cdot s\left(\delta ,t_{\text{min}},t_{\text{max}}\right)\cdot r\left(\epsilon ,t_{i-1},t\right)$$ (4)

Note that each factor can be “deactivated” by setting its parameter (*τ*, *δ*, or *ϵ*, respectively) to *∞*.

#### 2.2.4 Hard real-time (HRT)

Similar to FRT, the usefulness *u* is interpreted more strictly for this class. Violations of hard real-time constraints (*u* = 0) are considered severe incidents. The system is assumed to be in an undefined state. It may even be dangerous to itself and its environment. Such events must be detected and handled as quickly as possible to restore a stable situation and prevent any harm. This detection of violations is a key feature of µRT and is implemented using timers, which will trigger either a recovery attempt (can be defined for each component individually) or a system panic (system-wide default) exactly when a violation occurs.

Furthermore, µRT forbids HRT transmissions to be “overwritten,” which might result in the loss of a vital data point. As a result, further communication may be blocked as long as one or more HRT consumers have not processed previous messages yet (*cf.* section 2.6).

### 2.3 Design concepts

Now that the fundamental definitions of constraints and real-time classes have been presented, several particularities remain about how µRT achieves its real-time characteristics. Most importantly, µRT uses a sophisticated approach to track communication timestamps to detect violations of real-time constraints, which is detailed in section 2.3.2.

#### 2.3.1 Event-driven system design

µRT fully embraces the paradigm of event-driven architectures and avoids any periodic polling. This ensures that all events are handled as soon as possible with minimal latency and task prioritization and preemption are left to the scheduler of the underlying RTOS. Due to the absence of periodic “synchronization points,” jitter may increase, though, if the runtime complexity of some components varies greatly during operation. However, real-time software, in general, should be designed to exhibit consistent processing time in the first place. As a result, systems using µRT are more sensitive to bad implementations (concerning real-time characteristics), so developers will be encouraged to optimize such a code. Nevertheless, periodic execution of tasks with a given frequency remains desired in many situations, such as reading sensor data. With µRT, such behavior can be realized by means of periodic timers, which regularly fire with a specified frequency and emit events that eventually trigger task execution. This method is more elaborate as it involves an additional component (the timer) to achieve the time-triggered operation of the event-driven system, but that is actually intended. Because event-triggered task execution should be preferred in most situations, µRT deliberately encourages developers to follow this software design paradigm.

#### 2.3.2 Validation of timing constraints

Another important aspect of µRT is its approach to tracking latencies and validation of timing constraints at runtime, which are not defined per data point (e.g., sensor data have to be processed within a certain time frame), but by individual participants in the system (e.g., information must be received before it is older than *τ*). This approach effectively results in usability values per data point *and* consumer, so each participant can define and validate its individual timing constraints independently. Especially if additional stages are added to a data processing pipeline, making it more expensive regarding computation time, constraints of later stages remain valid and require no adjustment.

A crucial detail for this approach to work is correct tracking of the origin times of information, which is not the same as when the data are being transmitted within a system. While that data contains information, the latter emerges as soon as an event or state is observed and not only when it is encoded into some form of data structure, such that

$$t_{\text{info}}\leq t_{\text{data}}$$ (5)

holds. This definition is particularly important with regard to data processing pipelines, in which multiple components are arranged in a chain. After each stage in that pipeline, data is transmitted to the following component, and *t*_data,*i*_ increases continuously, whereas *t*_info,*i*_ remains unchanged.^3^ The benefits of this approach become obvious when analyzing the opposite case. If each component $k\in \left\{2,\ldots ,n\right\}$ in a data processing pipeline of length *n* would define its own relative deadline *τ*_*k*_ > 0 regarding its preceding stage, the overall deadline of the entire pipeline *τ*′ would be defined by

$$\tau^{\prime }\leq \overset{n}{\underset{k=2}{\sum }}\tau_{k}$$ (6)

The edge case of an equilibrium would only occur if all components fully exhaust their time budget: Δ*t*_*k*_ = *τ*_*k*_ *∀k*. As soon as any component requires less time (Δ*t*_*k*_ < *τ*_*k*_), the overall time budget *τ*′ is also reduced by that difference: *τ*′ ← *τ*′ − (*τ*_*k*_ − Δ*t*_*k*_). As a result, pipelines might miss *τ*′ because individual components are too fast, or all *τ*_*k*_ need to be increased to compensate for this effect, resulting in an overly optimistic initial value of *τ*′, which is no longer related to the actual use case. By referencing all timing constraints to the absolute origin time of information *t*_info_, the constraints of the entire pipeline are defined exactly by the last component, and each previous component defines the timing constraints of the pipeline up to that stage.

µRT specifies an information time *t*_info,*i*_ per data point *i* and validation of all timing constraints always refer to this value. For latency constraints, the point in time *t*_l,*i*_ at which the deadline *τ* is missed is hence defined by

$$t_{\text{l},i}=t_{\text{info},i}+\tau$$ (7)

and µRT can thus arm a timer to trigger as soon as *t*_l,*i*_ has elapsed, indicating a timing violation. The two critical times for synchronicity constraints, $t_{{\text{s}}_{\text{min}},i}$ and $t_{{\text{s}}_{\text{max}},i}$ , are likewise defined by

$$t_{{\text{s}}_{\text{min}},i}=t_{\text{max}}-\delta \;\text{and}$$ (8)

$$t_{{\text{s}}_{\text{max}},i}=t_{\text{min}}+\delta$$ (9)

While $t_{{\text{s}}_{\text{max}},i}$ is validated by means of a timer as well (actually only a single timer is required for latency and synchronicity validation; *cf.* Equation 12; sections 2.6 and 2.7), $t_{{\text{s}}_{\text{min}},i}$ is checked whenever data is retrieved by the consumer. If it was fetched too early, a timing violation has occurred. Regarding the rate, only the most critical constraint *ϵ*′ among all *n* consumers of a data source needs to be considered for the definition of the critical time *t*_r,*i*+1_:

$$t_{\text{r},i+1}=t_{\text{info},i}+\epsilon^{\prime }\;\text{with}$$ (10)

$$\epsilon^{\prime }=\text{min}\left(\left\{\epsilon_{1},\ldots ,\epsilon_{n}\right\}\right)$$ (11)

Hence, validation of *ϵ*′ is not performed every time data is fetched by a consumer, but only when it is provided by the producer. Therefore, rate validation does not require another timer for each consumer but only one per producer (at most; *cf.* section 2.6).

Due to these mechanics, some possible side effects should be kept in mind when working with µRT. When data is provided by a producer, the contained information might already violate the latency or synchronicity constraints of consumers:

$$t_{\text{data},i}>\text{min}\left(t_{\text{l},i},t_{{\text{s}}_{\text{max}},i}\right)$$ (12)

A similar effect occurs whenever the difference between *t*_info,*i*_ and *t*_data,*i*_ exceeds a rate constraint:

$$\begin{array}{cc} & t_{\text{data},i}-t_{\text{info},i}>\epsilon^{\prime }\\ \;\Leftrightarrow & t_{\text{data},i}>t_{\text{info},i}+\epsilon^{\prime }\\ \;\Leftrightarrow & t_{\text{data},i}>t_{\text{r},i+1} \end{array}$$ (13)

As a result, a timing violation is detected as soon as the data is committed. Strictly speaking, this is already too late because, in both cases, the critical point in time has already elapsed. For µRT’s validation mechanisms, there is obviously no way of knowing about such data before it exists. Although detection of timing violations might be delayed in such situations, µRT still acts as quickly as possible.

There is yet another possible edge case that should be considered when specifying the rate constraints of consumers. Because of Equation 5, µRT might detect a rate violation, although future data would provide valid information:

$$t_{\text{info},i+1}<t_{\text{r},i+1}<t_{\text{data},i+1}$$ (14)

Therefore, rate checks in µRT are rather conservative, and constraints *ϵ* should be specified with this in mind.

Finally, a last particularity of µRT’s timing validation mechanics is worth pointing out. As already mentioned, all constraints are defined by consumers, so in a sequential data processing pipeline, there are two steps for which no constraints can be defined: the observation, which initiates the pipeline, and the ultimate action at its end. In the former case, latency and jitter do not apply, and the rate can be validated by the subsequent component. However, for the latter case, the timing of the final action can only be validated by yet another consumer. The component that executes the action needs to provide information to confirm that the action has been conducted, and the additional component—also called *monitor*—consumes this information and validates its real-time behavior. While the use of such monitors seems complicated and inefficient at first glance, a single component can monitor all pipelines in a system, resulting in only minimal overhead.

#### 2.3.3 Concurrency and mutual exclusion

For control of concurrent access to shared data structures, µRT relies on mutex locks and condition variables. Although lock-free methods are generally to be preferred for real-time systems (Anderson et al., 1997), they are difficult to realize for many aspects of µRT, in particular without the C concurrency support library (stdatomic.h), which was only introduced with C11 (ISO, 2011) and would make µRT unusable for projects that do not support this version of the C standard. Especially in the context of MCUs, more conservative standards are often preferred (even Linux was only recently lifted to C11 from C89; Torvalds et al., 2022). Conversely, most MCUs feature only a single core anyway, such that the benefits of a lock-free implementation are rather limited.

#### 2.3.4 Configurability

In order to adapt µRT to the specific requirements of individual use cases, multiple feature flags and settings are provided to configure the implementation at compile time. First and foremost, the three subsystems—synchronization, publish–subscribe, and RPC (*cf.* Figure 2)—can each be enabled or disabled as required, and another global flag selects between debug and release builds. These settings are particularly useful if code size needs to be reduced to reduce ROM utilization. Selecting a release build also disables many sanity checks and improves performance tremendously.

There are several more settings regarding the two communication subsystems. On the one hand, tracking of profiling information can be enabled or disabled for each of the two subsystems. While such information can help track down bottlenecks, the logic obviously requires additional resources in ROM, RAM, and CPU time. On the other hand, the validation of timing constraints can be enabled or disabled as required *via* a total of five flags; for publish–subscribe validation of latency, synchronicity and rate constraints can be selected individually, and the same applies for latency and synchronicity constraints for RPC interaction. Even with all validation logic disabled, µRT still distinguishes the four real-time classes (*cf.* section 2.2), which is a perfectly legitimate use case. Once again, disabling these components saves resources in all three domains.

Further settings allow fine-tuning µRT even further by setting the sizes (i.e., number of bits) for several frequently used data types, such as temporal delays^4^ and identifiers for topics and services (*cf.* sections 2.6 and 2.7). There are also flags to select alternative algorithms for selected components of µRT, although these are not recommended for most scenarios and therefore are not discussed in this work. Finally, more settings allow configuring and interfacing OSAL, the abstraction layer for interaction with the operating system (*cf.* section 2.4).

### 2.4 Operating system abstraction layer

µRT defines its own abstraction layer to interface the underlying OS and event system. For applications using this middleware, it is recommended to stick to this API as well to ensure portability. The following features must be made available to µRT by mapping the according functions to OSAL.

• unique timestamps: Hardware timers in many MCUs feature only limited ranges (i.e., 16 or 32 bits) and tend to overflow frequently when setting the frequency to high values (e.g., 1 MHz). By definition, µRT uses timestamps at 1 µs resolution and requires the RTOS to provide an according accumulated system time or map a lower resolution time to µs equivalents.
• mutex locks and condition variables: Concurrent access to several components of µRT is prevented *via* mutex locks. Condition variables are used to inform nodes asynchronously about released locks.
• timers: µRT makes extensive use of timers to detect timing violations. Preferably the RTOS uses actual hardware timers so that violations result in the execution of an interrupt service routine (ISR) and according reactions are triggered as soon as possible.
• threads: OSAL defines a set of functions to control thread execution. Although thread handling may differ significantly between individual RTOSes, only very common functions are required by µRT.
• event system: µRT does not implement its own event system but relies on an externally provided implementation. Such can be part of the RTOS (as is the case for ChibiOS; di Sirio, 2020, 2022), or another system can be mapped to OSAL. µRT requires events to be emitted *via* broadcasts, and threads can be signaled individually.
• output: In order to print messages to output and error streams, the according functions must be provided. While in most cases, these will be aliases to the standard C function fprintf(), there are exceptions where this is not available (as is the case with AMiRo-OS).
• assert: When built with debug flags enabled, µRT performs many sanity checks in the form of assertions. As with the output functions, the OSAL assertion can be mapped directly to the standard C assert(). When further code (e.g., to stop a motor) shall be executed, such can be easily induced at this point.

### 2.5 Core components

As shown in Figure 2, the fundamental components of µRT comprise the *core* and *nodes* as well as an optional mechanism to synchronize *nodes*. As a central entity, the *core* is a static data structure, which exists exactly once and is globally available within a µRT context. It holds lists of all *nodes*, *topics*, and *services* and offers methods to control execution flow on a top level.

*Nodes* define the interface for the actual participants in the system. Each *node* is executed in its individual thread but may control further threads. The main() function for each *node thread* is part of the µRT implementation and subdivides the lifespan of each *node* into three phases, each of which can be interfaced *via* a custom callback function.

1. startup: This initialization phase is individual to each *node.* Before execution proceeds to the next stage, all *nodes* are synchronized by the *core.*
2. operation: Since µRT follows a strictly event-based approach, actions will only be performed if the *node thread* is triggered by some event source. Such triggers can be anything (e.g., communication, timers, or hardware interrupts) and are fully customizable. The only mandatory event belongs to the *core* in case of a shutdown request or a system panic.
3. shutdown: As soon as a *node* is requested to stop, it enters this final phase. The reason for the shutdown is propagated to all *nodes*, so appropriate actions can be executed.

In many situations, it is useful to synchronize multiple threads. This behavior is not trivial to realize by events only as each involved thread would require information about the others, resulting in a violation of the decoupling requirements (*cf.* section 1.2.1). To this end, µRT features *syncgroups* that can hold an arbitrary number of *syncnodes* ^5^ to be synchronized. Each thread that has joined a *syncgroup* can call a non-blocking synchronize() method at some point in time. If the result of that function call indicates that some *syncnodes* of the *syncgroup* have not synchronized yet, the calling thread has to wait for a synchronization event. As soon as the final *syncnode* executes that method, this event is emitted to the entire *syncgroup*, except for the calling thread, which receives an according return value. For use cases where the µRT synchronization mechanism is not desired, it can be disabled entirely *via* a feature flag. If a system comprises multiple µRT contexts (*cf.* section 2.8), these mechanics can also be employed to synchronize all *nodes* in the entire system. This is done once by default to make all *nodes* enter the operation phase simultaneously.

### 2.6 Publish–subscribe

**FIGURE 3:**
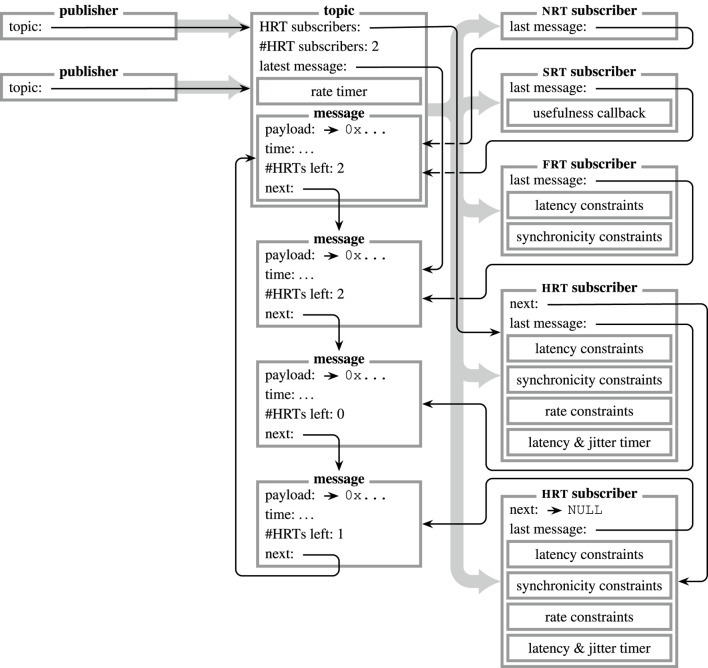
Visualization of a publish–subscribe interaction in µRT. Information is provided by an arbitrary number of *publishers* and passed *via* a *topic* to an arbitrary number of *subscribers* (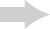). 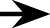
represent aggregations (i.e., pointers in C). Data are buffered by the *topic* in a ring buffer of *messages* of arbitrary size ≥1 . Each message holds a counter of remaining *HRT subscribers*, so the *topic* will only reuse a *message* if its counter is 0. While each subscriber class holds different members, all track the last message consumed. *HRT subscribers* are also arranged in a list, starting at the *topic* and ordered by their rate constraints. In the depicted situation, only one more *message* can be published before the upper *HRT subscriber* has to fetch the lowermost *message*.

For unidirectional communication, *publishers* provide information anonymously through *topics*, which act as mediators and inform all registered *subscribers* (*cf.* Figure 3). In order to retrieve a *topic* by its identifier (µRT uses no strings but numerical values to identify *topics*), the *core* provides the according methods to search among all available topics.^6^ While *publishers* are registered to a *topic* at initialization, *subscribers* can subscribe and unsubscribe dynamically during operation. Every *topic* holds one or more *messages* in a ring buffer, each of which holds a custom payload structure to carry data. This buffer is implemented as a distributed list instead of a contiguous array. Therefore, further elements can be added by any component at any point in time.

The timestamp of each *message* is of major importance. As described in section 2.3.2, it does not describe the point in time when the *message* was published (*t*_data_), but the origin time of the contained information (*t*_info_). Due to this differentiation, the *message* buffer of each *topic* is not ordered by *t*_data_ but by *t*_info_, so that the latest element in the buffer will always be the *message* with the latest information. Conversely, when a new *message* that carries older information is published, it is not appended but rather enqueued according to its *t*_info_ value.

Whenever information is published, several steps are executed by µRT:

1. The *publisher* requests a *message* from the *topic.* If no *message* is available due to pending *HRT subscribers* (*cf.* section 2.2) or if the oldest element in the buffer already holds more recent information, the publish attempt fails. Otherwise, the metadata of the *message*, such as information time, is updated, and payload data is copied to its buffer. After that, the *message* is enqueued again in the *topic*’s buffer, according to its *t*_info_.
2. Metadata at the *topic* is updated. If the published *message* is the latest one in the buffer, the *topic*’s rate timer is re-armed according to the timestamp of the new *message* and the most critical rate constraint among all registered *HRT subscribers* *ϵ*′. Each *topic* also holds a list of all registered *HRT subscribers*, so their timers to detect latency or jitter violations are updated as well. Finally, an event is emitted to inform all registered *subscribers* about the new *message.*
3. A registered *subscriber* can fetch the next *message* from the buffer and copy its payload. In case multiple *messages* have been published since the last iteration of this *subscriber*, those can either be fetched subsequently, or the *subscriber* can fetch the latest *message* directly. For *HRT subscribers*, the timer is updated or deactivated if there are no further pending *messages. SRT subscribers* and *FRT subscribers* can calculate the usefulness *u* of the fetched *message* (*cf.* section 2.2).

Each *HRT subscriber* only needs to validate latency and synchronicity constraints individually, whereas rate constraints are validated by the *topic* for all registered *HRT subscribers*.

The described approach completely omits dynamic memory allocation, as all buffers are static. While this is beneficial for temporal consistency, each information transfer requires the data to be copied two times. First, the *publisher* writes to the *message* payload. Second, each *subscriber* has to copy that buffer when fetching the *message*. Although this will have a significant performance impact for increasing amounts of data, this approach optimizes µRT for determinism and predictability. Computational complexity scales linearly with the number of *messages* in the buffer *m*, the payload *p*, and the number of *HRT subscribers* *s*_HRT_ registered to the *topic*:

$$O\left(\alpha \cdot m+\beta \cdot 2p+\gamma \cdot s_{\text{HRT}}\right)$$ (15)

The weight factors *α*, *β*, and *γ* are unknown but will be relevant for performance evaluation in section 3.2.2. Finally, producers and consumers are decoupled in space, time, and synchronization.

### 2.7 Remote procedure calls

**FIGURE 4:**
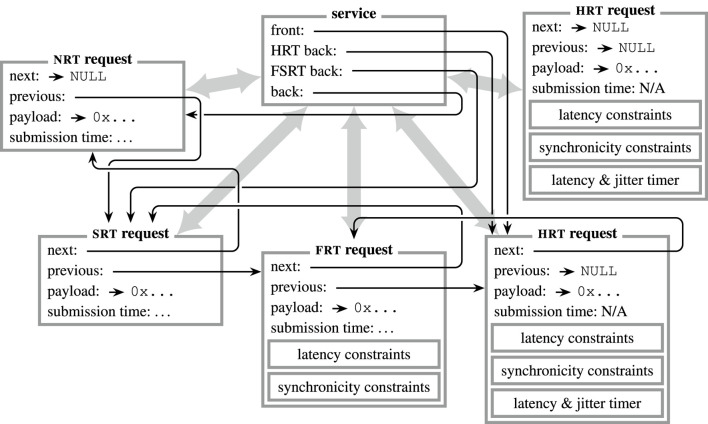
Visualization of an RPC interaction in µRT. Information is passed from *request* to *service* on submission and *vice versa* on response (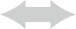). 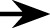
represent aggregations (i.e., pointers in C). An arbitrary number of *requests* may be submitted to a *service*, which buffers those in a queue, ordered by real-time class and constraints. The servicing thread dispatches one *request* at a time from the front of that queue, processes it, and returns a response (by reusing the original *request* instance). In the depicted situation, four *requests* are already enqueued at the *service*, whereas another *HRT request* is available but currently not in use.

The basic idea of RPCs is to trigger an action similar to a local function call but remotely at another component in the system architecture. Although this behavior can be achieved *via* publish–subscribe using a “request topic” and another “response topic,” this approach is inefficient because two *m*-to-*n* communication channels are used to emulate a single 1-to-1 interaction, and *services* would have no priority information about *requests*. Therefore, the RPC subsystem of µRT implements *request queues* at the *service*, which are ordered by real-time class and timing constraints (*cf.* Figure 4). Like *topics*, *services* are identified by numerical values and can be retrieved *via* the core.^7^ µRT employs a combination of locking and ownership mechanics to acquire *requests* and pass them between requesting and servicing threads. Each interaction is again subdivided into several steps:

1. A *request* must be acquired to be used for only one RPC interaction at a time. After a successful acquisition, the *request* is “locked” and “owned” by the requesting thread. Similar to publish–subscribe, metadata is updated and argument data is copied to the payload buffer.
2. The *request* is submitted to the *service*, and an event is specified to inform the requesting thread of completion. As part of the submission procedure, ownership is handed over to the *service*, and the *request* is enqueued at the *service* according to its real-time class and constraints. Eventually, the thread providing the *service* is informed *via* an event.
3. The servicing thread dispatches one *request* at a time from the *service*’s queue, thereby copying argument data and releasing its locked state.
4. As soon as the *service* is done, it tries to re-acquire the dispatched *request.* On success, it is locked again, any return values are copied to its payload buffer, and the requesting thread is informed *via* the previously specified event.
5. The requesting thread can retrieve the *request* and take over ownership again. For *SRT requests* and *FRT requests*, the usefulness *u* can be calculated at this point (*cf.* section 2.2).
6. As soon as all return data has been processed or copied, the *request* has to be released to finalize the interaction. Afterward, the *request* is unlocked and not owned by anyone anymore and hence is available again for further interactions.

The interplay of locking and ownership mechanisms plays a key role in this procedure. In contrast to *messages*, *requests* are not associated with a *service* for the lifetime of the latter but only during an ongoing interaction. This allows using a single *request* for interaction with multiple *services*. The downside of this approach is that a *request* could “get stuck” in the queue of a *service*, not being served for a long time and thus not being available for other, potentially more important interactions, which might compromise the responsiveness of a system and hence its real-time capability. In order to solve this issue, the two mechanics have been employed, and the following rules apply for handling *requests*.

• A *request* is available if it has no owner and is not locked.
• A *service* may only act on a *request* if it is locked and owned by the *service.*
• The requesting thread may retrieve a previously submitted *request* at any time, as long as it is not locked (by the *service*).

As a result, a submitted *request* can be aborted anytime and reused if needed. Although the servicing thread is not notified about this termination and will keep processing an already dispatched *request*, re-acquisition to return a response will fail. Because the canceled *request* might be used for the same *service* again and *services* cannot distinguish whether a *request* has already been dispatched or was just submitted but not dispatched yet, just checking ownership and lock state does not suffice. Therefore, the *request*’s submission time is saved on dispatch and used for comparison during re-acquisition before returning a response. If the timestamps do not match, re-acquisition fails, results are discarded, and no response event is emitted. However, it is often the case that neither return data nor a notification on completion is desired by the requesting thread in the first place. To this end, *requests* can be flagged as “fire-and-forget” by not specifying a response event on submission.

In contrast to the publish–subscribe interaction, there are no rate constraints for RPCs, so each *HRT request* validates its individual latency and synchronicity constraints by itself. Moreover, the timestamp each *request* holds describes the time when it was submitted to the *service* and is not related to the content of the payload as was the case for *messages*, so *t*_info_ = *t*_data_ always holds. The rationale behind these design choices is that RPCs should not be used for periodic communication within data processing pipelines but rather for sporadic or regular events to interact with such pipelines (*cf.* Figure 12). As mentioned previously, the *request* queue of each *service* is ordered and therefore subdivided into three parts. *HRT requests* are always inserted in the front part of the queue, ordered by their timing constraints, such that the most critical *HRT request* is served first. *FRT* and *SRT requests* are placed in the middle part of the queue, following the “first come, first serve” principle. The same applies to *NRT requests*, which are always appended at the very end of the queue.

Once again, all involved data structures are static, and no dynamic memory allocation is required. As with publish–subscribe, the downside is multiple copy operations. However, this approach allows all involved threads to be responsive to other events at all times and thus prevents stalling (i.e., deadlocks) by design. Computational complexity scales linearly with the queue length *q*, the payload *p*, and the number of enqueued *HRT requests* *r*_HRT_:

$$O\left(\alpha \cdot q+\beta \cdot 4p+\gamma \cdot \left(r_{\text{HRT}}-1\right)\right)$$ (16)

As with publish–subscribe, the weight factors *α*, *β*, and *γ* are unknown. *r*_HRT_ is reduced by 1 because the case to “enqueue” a single *HRT request* is trivial, so this term is only relevant for *r*_HRT_ > 1. Requesting and servicing threads are decoupled in space, time, and synchronization.

### 2.8 Platform-level interaction

All concepts of µRT so far only apply locally within a single process. On the one hand, this approach allows for fast information transfer *via* shared memory and simplifies several aspects, as details such as endianness and (de)serialization of data are of no concern. Especially in the context of MCUs, this is sufficient because many RTOSes do not feature strictly separated processes, and most MCUs do not even host an MPU (memory protection unit), which is fundamentally required to (efficiently) facilitate separated regions in memory. On the other hand, network communication is essential for modular systems and thus has to be considered. The major challenge in this regard is the vast variety of transports (e.g., Ethernet, SPI, UART, CAN, or FlexRay); protocols (e.g., TCP for Ethernet or TTCAN for CAN); and higher-level data distribution services (DDS). Hence, many solutions, such as ROS, ROS 2, MQTT, and RSB, only support selected interfaces (Stanford-Clark and Hunkeler, 1999; Quigley et al., 2009; Wienke and Wrede, 2011; OSRF, 2020; OSRF, 2022b) and may have even further restrictions (e.g., DDSes supported by ROS 2).

**FIGURE 5:**
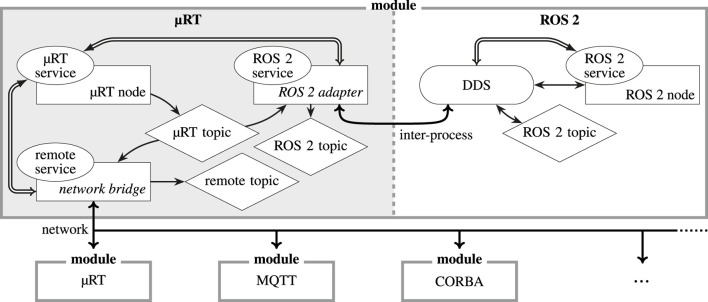
Example setup in a complex system with multiple hardware modules and various middlewares (only the most relevant interactions are depicted). Publish–subscribe interaction is indicated by 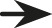, 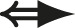
represent RPC interactions, and 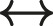
depict network and inter-process communication. µRT’s transport agnostic communication is realized by *bridge nodes*, such as “*network bridge”* and “*ROS 2 adapter”* in the figure. While the former has to (de)serialize *messages* and *requests* from/to a common format as defined for that network channel, the latter needs to translate them according to the employed DDS. The *bridge nodes* can provide an arbitrary subset of the remote/foreign *topics* and *services* they connect with to the local µRT instance and *vice versa*. If the network supports real-time communication (e.g., CAN), all modules running µRT form a distributed, real-time capable union in which network communication is completely transparent to all *nodes* (except *bridge nodes*, of course).

µRT approaches this challenge by not supporting any transport out-of-the-box but commits these tasks to *bridge nodes*. Such *nodes* can implement any transport and protocol and even filter messages if bandwidth is too limited to communicate all information. This method allows for integrating µRT in existing platforms, which already employ other middlewares, as depicted in Figure 5, and even further transports can also be supported.

There are two reasons for this design choice. First, several established solutions are available, and there is no need to develop yet another one. Instead, µRT aims to be used alongside traditional middlewares to enable the modularization of the real-time software of a system. Second, while TCP and UDP have been established as the *de facto* standard protocols for most communication in modern applications, there is no common standard for embedded designs and MCUs. Such devices are typically not powerful enough for these protocols, so less demanding technologies are employed, which are numerous and have differing properties. A limitation to a subset of these interfaces and protocols would therewith render µRT unsuitable for many use cases. As every module in a system may host different hardware with different capabilities and real-time requirements also vary significantly between use cases, µRT opts for this more general method.

The benefits of this approach are its flexibility and technology independence. Therefore, µRT can interface any communication channel. Moreover, as long as such an interface supports real-time communication, multiple µRT instances can form a distributed union in which all *topics* and *services* are accessible by all *nodes*, making the entire network effectively a single virtual µRT instance. This method has some drawbacks, as the additional work required to develop *bridge nodes* is less convenient and presents an initial hurdle. However, the intention is as follows: once a *bridge node* for an interface has been developed, it can be provided to the community and other developers can henceforth utilize it with minimal effort and enhance it as required.

Another potential pitfall when combining µRT with other middlewares is the absence of any discovery and advertising mechanisms. Such interaction schemes are commonly employed by other solutions to inform the system about newly created topics and services. Due to the strict real-time requirements of µRT, all such objects must be available as soon as system initialization is completed and may not be removed until shutdown. This may result in situations where a remote *topic* or *service* is available on the µRT side, although it has not been initialized yet (or has been removed again) within the foreign middleware, thus violating assumptions about the state of the communication network. Bridge nodes can solve this issue to some degree by providing a *topic* or *service* only after the advertising message has been received. The opposite case, which is removing a *topic* or *service*, is not possible, though.

## 3 Evaluation

µRT has been evaluated in three ways to compare it to existing middlewares. First, a qualitative comparison of features is given with respect to R2P and ROS 2 (*cf.* section 3.1), and performance data are presented thereafter in section 3.2. Because middlewares are fundamentally only a means to an end to ease software development, usability for developers is an essential aspect. Therefore, a study has been conducted and described in detail, and results are presented in section 3.3.

### 3.1 Features

For a qualitative comparison of features, the two most important competitors among the plethora of existing middlewares have been selected: ROS 2 due to its popularity in robotics and its ambitions to support real-time computing and R2P because it had been developed with similar goals in mind as for µRT. Table 1 lists ten important middleware aspects concerning the target application of µRT.

**TABLE 1:** Feature comparison of µRT, R2P (Migliavacca, 2013), and ROS 2 (Macenski et al., 2022).

Feature	ROS 2	R2P	µRT
Interaction schemes	Publish–subscribe	Publish–subscribe	Publish–subscribe
	RPC		RPC
	actions		
Decoupling	Space	Space	Space
	Time	Time	Time
	Synchronization	Synchronization	Synchronization
Real-time capability	Up to soft	Hard	Hard
	(depends on DDS)	(system-wide)	(system-wide)
Real-time classes	NA	Hard, soft, and non	Hard, firm, soft, and non
		(by RTCAN)	
Validation of	×	×	Latency
timing constraints			Synchronicity
			rate
All static memory	×	×	*✓*
Zero-copy communication	*✓*	*✓*	×
QoS	*✓*	×	*✓*
			(If profiling enabled)
Suitable for MCUs	×	*✓*	*✓*
Programming languages	C**++**	C**++**	C
	Python		
	…		

First, while R2P supports publish–subscribe interaction only, ROS 2 not only offers RPCs like µRT but also provides an additional “actions” interaction scheme. Actions are similar to RPCs but intended for long-running tasks. In short, an action is initiated by a client and the action server provides periodic feedback until the goal has been reached. Because such use cases should not require real-time capabilities in itself (underlying processes might, though), supporting actions was no goal for µRT. If such behavior is desired, it can still be realized using a service to initiate execution and a topic to provide periodic feedback or another service to provide feedback on demand.^8^ Concerning decoupling, all three middlewares are decoupled in all three domains.

However, when it comes to real-time capabilities, it is obvious that complete real-time support is not the primary focus of ROS 2. It cannot provide stricter than soft real-time and does not differentiate real-time levels at all. Conversely, R2P and µRT allow for system-wide hard real-time constraints and define multiple real-time classes with µRT even specifying an additional fourth class. Probably, the most distinguishing feature of µRT is its capability to validate real-time constraints at runtime. To the best of our knowledge, ROS 2 and R2P do not offer this functionality, nor does any other middleware.

An important aspect of real-time capabilities is memory management, more precisely, the absence of dynamic allocation. R2P still relies on memory pools, which are an optimized form of dynamic memory management but still include dynamic allocation. Therefore, only µRT manages to completely omit dynamic memory. The downside of this approach is that interaction in µRT involves payload data being copied multiple times. Conversely, ROS 2 and R2P allow for zero-copy communication.

Finally, ROS 2 and µRT provide QoS statistics, whereas R2P does not. Although ROS 2 can be used on MCUs, doing so comes with several limitations (*cf.* section 1.1.4). Hence, only R2P and µRT are considered suitable for such restricted platforms. The different focus on target platforms also shows in the (primarily) supported programming languages. µRT is completely written in C and only supports this language so far.^9^ For R2P, it is the same, but with C**++** instead of C. ROS 2, however, provides APIs for multiple languages (first and foremost C**++** and Python but also C) and thus offers the highest flexibility for developers.

Overall, this comparison shows the different focus areas of the three middlewares. Although ROS 2 primarily focuses on high-level software and offers many conveniences for developers of such but supports real-time computing and MCUs only subordinate, R2P and µRT aim at exactly these use cases. Again, µRT surpasses R2P in almost every aspect, except for zero-copy communication, in which µRT trades for uncompromising real-time capability.

### 3.2 Performance

The performance of µRT has been evaluated in terms of memory requirements and runtime performance scaling. Results regarding the former are presented in section 3.2.1, and scaling benchmarks are presented in section 3.2.2.

#### 3.2.1 Memory utilization

The memory footprint of µRT is of major importance as the middleware is targeted to be used on (32-bit) MCUs, for which available resources are very limited. The sizes of the integrated flash memory of such devices typically range from 16 to 1,024 kB, so compiled images need to be rather compact. System memory is even more scarce, with entry-level products featuring no more than 8 kB of RAM and only the most powerful devices exceeding 256 kB. Therefore, it is a crucial requirement for µRT to exhibit a small footprint in both regards. Therefore, it can be deployed on a wide range of MCUs.

Table 2 shows the sizes of all major components of µRT in system memory and compares them to R2P, as presented by Migliavacca (2013). Most noticeably, many rows in the table contain no values for R2P at all due to the lack of such components because R2P does feature neither synchronization mechanisms nor RPC interaction. Compared to µRT, it also omits a core component. Because there is only a single core per instance, these 37 bytes should be negligible in most scenarios. Other than that, nodes are about 50% larger for µRT, which is a significant increase, but with an absolute value of 56  B, the footprint is still considered reasonable. Both components of the synchronization subsystem are acceptably small, with no more than 32 B. Publishers are much smaller for µRT and require only 4 B (25% compared to R2P), as they essentially hold just a pointer to the topic. Topics are also larger for µRT than they are with R2P. Especially when QoS is enabled, the memory footprint is 157% larger, but even without QoS, the increase is still 57%. It should be noted that each topic in µRT already holds a message, so those 32 B need to be subtracted for an apples-to-apples comparison, resulting in a somewhat smaller increase of 100% with QoS enabled and even the same 56 B as for R2P with QoS disabled. The size of subscribers in µRT depends on their real-time class (*cf.* section 2.2) but is estimated in Table 2 with the upper bounds. While they are slightly smaller when QoS is disabled (92%), size increases by a factor of 2 when enabled. Finally, the footprints of RPC components are similar to those of the publish–subscribe subsystem. Requests are again much larger when QoS is enabled, with an even starker difference of 88 B (157%). Conversely, services are of constant size, with 48 B acceptably small. Overall, the memory footprints of µRT’s components are about 50%–150% larger than their pendants in R2P, but absolute values remain small enough to be reasonable even for entry-level MCUs.

**TABLE 2:** Memory footprints of the most important components of µRT and R2P (Migliavacca, 2013). For these measurements, µRT has been configured to reasonable settings, e.g., identifier sizes for *topics* and *services* were set to 16 bits. All values are given in bytes.

Subsystem	Component	µRT	µRT (no QoS)	R2P
Core	Core	37	37	–
	Node	56	56	36
Synchronization	Syncgroup	28	28	–
	Syncnode	32	32	–
Publish–subscribe	Publisher	4	4	16
	Topic	144+ messages	88+ messages	56
	Subscriber	≤136	≤44	48+ messages
	Message	32+ payload	32+ payload	Payload
RPC	Request	≤144+payload	≤56+payload	–
	Service	48	48	–

When considering the required amount of flash memory, µRT can scale from a modest 2.2 kB to 18.2 kB due to its high configurability (*cf.* section 2.3.4). Unfortunately, a direct comparison with R2P is difficult to achieve because Migliavacca (2013) only stated values, including the RTOS; hence, such was not conducted for this work. Absolute footprint sizes of µRT’s core, the three subsystems, and a complete configuration are given in Table 3 and represent the worst-case scenario among all evaluated MCUs.^10^ When comparing memory footprints in the binary image, the publish–subscribe subsystem is the most expensive component, with RPC close behind. Conversely, synchronization functionality has a minor impact of no more than 1 kB, even for debug builds. When comparing release and debug scenarios, footprints increase between 49% and 95% for the latter (67% on average). Enabling QoS is less expensive, with only 13%–50% larger footprints (29% on average). In both cases, impacts are most pronounced for the publish–subscribe subsystem. Considering the required flash memory exceeding 16 kB with everything enabled and considering that a sophisticated RTOS is required, µRT is hardly a viable option for MCUs with 32 kB of flash or even less. Based on our experience, a minimum of 128 kB is recommended for development (debug builds) and 64 kB for deployment (release builds), even with optimizations (i.e., garbage collection and link time optimization) enabled.

**TABLE 3:** Memory footprint of µRT in a compiled image. Results have been obtained on a STM32-F405RG (32-bit Arm Cortex-M4), for which the values were the largest among all evaluated MCUs.^10^ Code has been compiled using GCC 11.3.1 (Arm GNU Toolchain) with optimizations such as garbage collection (GC) and link time optimization (LTO) disabled. Further settings of µRT have been configured to reasonable values; for e.g., identifier sizes for *topics* and *services* were set to 16 bits and profiling was enabled. All values are given in bytes.

	Debug build		Release build	
	QoS enabled	QoS disabled	QoS enabled	QoS disabled
Core	3,384		2,264	
Synchronization	1,024		640	
Publish–subscribe	7,952	6,224	4,800	3,200
RPC	6,296	5,592	3,952	3,184
Complete	18,648	16,216	11,520	9,216

#### 3.2.2 Runtime performance and scaling

In order to evaluate the runtime performance of µRT, an extensive set of benchmarks has been conducted.^11^ These benchmarks have been designed in a way that scaling effects for each middleware component can be assessed, as well as differences between real-time classes. Therefore, scenarios have been implemented carefully to represent worst-case situations. Thread priorities were set in a way that nodes would constantly block each other, and the MCUs were continuously stressed by an additional low-priority thread. Moreover, each benchmark was repeated 1,000 times, so meaningful minimum and maximum values could be obtained to assess synchronicity performance.

**FIGURE 6:**
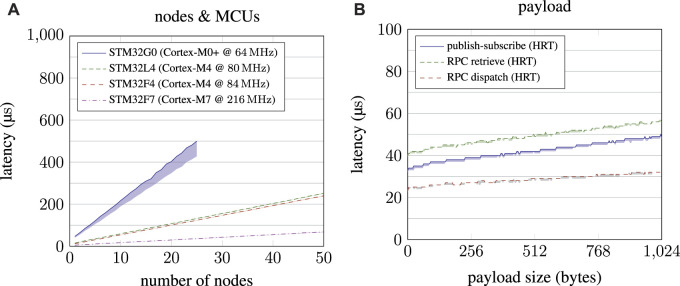
General performance measurements of µRT. On the left-hand side **(A)**, performance scaling when increasing the total number of nodes in a system is depicted, thereby comparing different MCUs. On the right-hand side **(B)**, the performance impact of payload sizes is shown for both communication schemes supported by µRT.

The graphs in this section depict only the results of the worst-performing component (e.g., the node with the highest latency). Note that each graph consists of a line and a shaded area “hanging” below. Although the former represents the largest measured values, the latter depicts the range between the lowest and highest values in the result data. However, for most graphs, the shaded area is barely visible. Except for the data shown in Figure 6A, all results were obtained using an STM32L476RG (Cortex-M4 @ 80 MHz).

First, performance scales linearly with the number of nodes in a system, as shown in Figure 6A. For this benchmark, a single timer triggered all nodes, which just measured the latency until the event was eventually processed. As expected, absolute performance varies significantly between different MCUs. Although most MCUs demonstrate consistent performance, the STM32G0 exhibits significant temporal variance, which is most probably caused by its Cortex-M0+ core.

**FIGURE 7:**
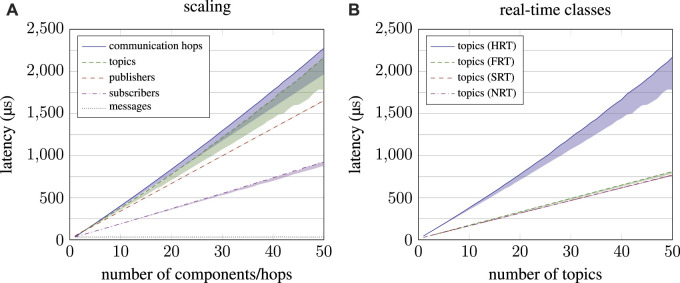
In-depth performance analysis of the publish–subscribe subsystem. On the left-hand side **(A)**, performance scaling when increasing the number of components in a system is depicted. On the right-hand side **(B)**, the performance of the four real-time classes is compared for increasing number of topics.

**FIGURE 8:**
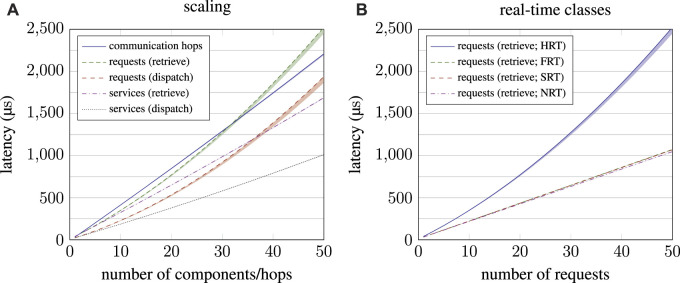
In-depth performance analysis of the RPC subsystem. On the left-hand side **(A)**, performance scaling when increasing the number of components in a system is depicted. On the right-hand side **(B)**, the performance of the four real-time classes is compared for an increasing number of requests.

Figure 6B depicts performance scaling for publish–subscribe and RPC interaction with increasing payload sizes. Again, performance scales linearly, as expected. More interestingly, the first communication of an RPC interaction (until the service dispatched the request) is significantly faster than publish–subscribe, although the return communication (until the answered request is retrieved again) takes longer overall. This finding indicates that fire-and-forget requests should be used whenever possible. The graphs also show the potential performance gains if zero-copy communication was possible with µRT. Especially when comparing the time scales with Figures 7, 8, copy operations have only a minor impact on the overall performance.

For benchmark results of the publish–subscribe subsystem, as depicted in Figure 7, two striking features catch the eye. First, for an increasing number of communication hops, topics and (HRT) subscribers, synchronicity characteristics deteriorate. A closer look at the data reveals that only the very first interaction is significantly faster (by 13.2% for topics), and excluding this data point results in a jitter of 1.2% instead of 12.2% regarding topics. Second, performance graphs of communication hops and topics do not scale linearly but slightly exponentially. Again, this is only the case for HRT interaction, whereas performance scales linearly for all other real-time classes. When recalling the estimated complexity of publish–subscribe interaction from Equation 15, the reason becomes obvious. Neither the number of hops nor the number of topics can be increased without increasing the number of subscribers *s*_HRT_. Doing so effectively results in a complexity multiplied by *s*_HRT_ and, therefore, a quadratic increase in latency because validation timers of all HRT subscribers need to be updated with every interaction:

$$\begin{array}{cc} & s_{\text{HRT}}\cdot O\left(\alpha \cdot m+\beta \cdot 2p+\gamma \cdot s_{\text{HRT}}\right)\\ = & \;O\left(s_{\text{HRT}}\cdot \left(\alpha \cdot m+\beta \cdot 2p+\gamma \cdot s_{\text{HRT}}\right)\right)\\ = & \;O\left(\alpha \cdot m\cdot s_{\text{HRT}}+\beta \cdot 2p\cdot s_{\text{HRT}}+\gamma \cdot s_{\text{HRT}}^{2}\right) \end{array}$$ (17)

Nonlinear performance scaling for HRT communication is an accepted trade-off made by µRT, though, in order to validate timing constraints for such critical components. Moreover, the rather low expression of exponential scaling in the data suggests that the corresponding weight *γ* is rather small compared to *α* and *β*. Figure 7A shows constant performance for an increasing number of messages. This benchmark does not represent the worst-case scenario, as *t*_info_ increased with every interaction, so messages did not have to be enqueued (general case; linear complexity) but could just be appended (constant complexity). With this in mind, the presented graph must be interpreted as typical performance rather than worst-case performance, which scales linearly.

A similar picture emerges for the RPC subsystem. Performance evaluation, as presented in Figure 8, apparently shows the same effects as the publish–subscribe, but more pronounced. Again, recalling the complexity estimation from Equation 16 reveals the cause. As with HRT subscribers for publish–subscribe interaction, increasing the number of HRT requests *r*_HRT_ effectively results in quadratic scaling regarding this factor:

$$\begin{array}{cc} & r_{\text{HRT}}\cdot O\left(\alpha \cdot q+\beta \cdot 4p+\gamma \cdot \left(r_{\text{HRT}}-1\right)\right)\\ = & \;O\left(r_{\text{HRT}}\cdot \left(\alpha \cdot q+\beta \cdot 4p+\gamma \cdot \left(r_{\text{HRT}}-1\right)\right)\right)\\ = & \;O\left(\alpha \cdot q\cdot r_{\text{HRT}}+\beta \cdot 4p\cdot r_{\text{HRT}}+\gamma \cdot \left(r_{\text{HRT}}^{2}-r_{\text{HRT}}\right)\right) \end{array}$$ (18)

Notably, performance does not scale exponentially when increasing number of communication hops this time because there is only a single request per hop (*r*_HRT_ = 1), so the hindmost term has no effect in this case. Although exponential effects are much more pronounced for RPC than was the case for publish–subscribe, the weighting factor *γ* remains relatively small.

Overall, performance figures confirm the overall linear scaling of µRT, although some scenarios exhibit exponential latency increase. However, this is only the case for HRT communication, and even then, nonlinearity is little pronounced for scales reasonable for MCUs. Especially when considering the findings concerning RPC interaction, request queues with lots of HRT requests should be avoided in the first place for the sake of a responsive system.

### 3.3 Usability

In order to assess the usability of µRT for software developers, a study has been conducted. The goal of this study was not just to evaluate the ease of use of µRT by itself because this information alone would not be meaningful, but how it fares compared to ROS. It was conducted as part of the exercises for the lecture “Autonomous Systems Engineering” at Bielefeld University and carried out in multiple weekly sessions. This way, a comprehensive set of introductory information, programming tasks, and questionnaires could be used in the study. In section 3.3.1, the study design and applied methods are described in detail before the results are presented afterward in section 3.3.2.

#### 3.3.1 Study design

First, a within-person design was chosen for the study, and it was subdivided into four parts, conducted in weekly sessions of 2 h each.

1. ex ante: Participants were asked to fill in a questionnaire about demographic information and prior experience in various areas of computer science. For the latter, six-level Likert scales were used with the available options “none,” “novice,” “advanced beginner,” “competent,” “proficient,” and “expert.”
2. ROS line following: In the first session, an introductory lecture was given, explaining fundamental concepts of middlewares in general and specifically ROS. In the second session, participants were given the task of making AMiRo follow a line in a simulation environment using ROS (C**++** only), for which they were allowed two sessions to complete. Afterward, participants were asked to complete a questionnaire to assess their experience.
3. µRT tutorial: This time, no introductory lecture was provided, but participants were instructed to work through a tutorial on µRT instead and to fill in another questionnaire afterward. Participants were allowed to spend two sessions with this part.
4. µRT line following: Another task was given to make AMiRo follow a line, but this time using µRT on a real robot, and again participants were asked to rate their experience thereafter by means of another questionnaire. As with the other tasks, participants could spend two sessions on this one.

Questionnaires for the three tasks were identical, each consisting primarily of a USE questionnaire as defined by Lund (2001), but supplemented by three further questions—level of completion (self-estimation), required time, and the number of requests for assistance—as well as corresponding free-text fields to account for qualitative responses. Obviously, the results of those questionnaires are the dependent variables in this study.

The independent variable of interest was the employed middleware—ROS *versus* µRT—so the two most important parts of the study are the line-following tasks. The decision was made against a between-person design because the scenarios could not match each other exactly. As ROS cannot be run on the MCUs of AMiRo, that task had to be conducted in a simulation environment rather than an actual robot. Conversely, no simulator integration for µRT exists so far, so these scenario differences were inevitable. Furthermore, the acquisition of participants for a study of this scale is difficult, especially during the COVID-19 pandemic, making those a very scarce “resource.” The information gained per participant hence needed to be maximized. Moreover, the in-person design with this order—ROS first and µRT thereafter—does not compromise the validity of this study’s results because many software developers in robotics are already experienced with ROS and would use µRT as supplemental middleware on the real-time level of a system. Hence, the study design represents real use case scenarios very well.

Each task of this study was designed so it could be completed in about 90 min, but participants were permitted up to 4 h. For optimal comparability, both line-following tasks were designed identically: participants had to create a new node, add a subscriber to receive sensor information from a prepared topic, write a simple logic to calculate a two-dimensional motion vector, and interface an existing service to make AMiRo move. The quality of the implemented line-following algorithm was of no concern for this study, as the focus was on understanding middleware concepts and the ability to apply these to actual implementation.

#### 3.3.2 Questionnaire results

In total, 21 students in computer science participated in the study. Ages ranged 21–28 (median 23.5) years. Of the 21 students, thirteen were male and six were female participants, and two were not specified. All students had a bachelor’s degree except one who had no academic degree yet. Thirteen (62%) participants had already worked with ROS before, and seven (33%) had experience in robotics, although self-assessed skill levels ranged no higher than “competent.” The same applies to experience in the two relevant programming languages, C**++** and C, whereby the mean expertise for the former was about 1/2 skill level higher. Notably, experience in Python was much higher, with the median at “competent.” This result confirms the decision to restrict the ROS task to C**++** because the strongly differing programming skills might have influenced the study results. Finally, although 11 (52%) participants had worked with MCUs before, none stated any expertise with RTOSes, indicating that the previous experiences were made in bare-metal programming of MCUs instead of using sophisticated RTOS software environments.

Unfortunately, although all students did work on all tasks, not all questionnaires were completed. Of the 21 participants, only 12 (57%) assessed the ROS line-following task, 10 (48%) submitted valid questionnaires for the µRT tutorial, and 6 (29%) rated the µRT line-following task. Moreover, complete data about all three tasks are available for just five students (24%). For this reason, results will be distinguished hereafter by whether they were obtained from the entire cohort or only from the five participants who provided complete information. Those two subsets of the cohort will be referred to as group A and group B.

**FIGURE 9:**
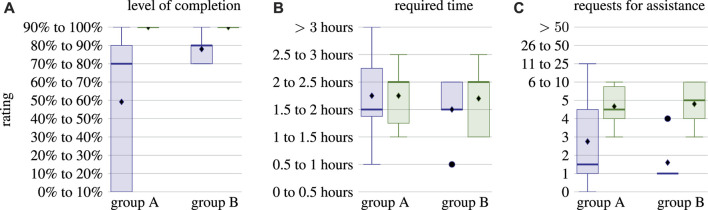
Statistical evaluation of the three additional questions of the questionnaires: self-estimated level of completion **(A)**, required time **(B)**, and number of requests for assistance **(C)**. Each pair of box plots depicts results for ROS ((left)) () and µRT (right) (), respectively. Each axis shows results for the entire cohort (group A) and only those participants who filled in the questionnaires about ROS and µRT (group B). Vertical axes are labeled by the options participants had to choose from, as they were available in the questionnaires.

Before examining the results of the USE questionnaires, the evaluation of the three additional questions provides insights into the correlation between participants who only filled in the questionnaires about ROS (group A) and who also provided information about their experience with µRT (group B). Figure 9 shows that the ROS task completion level varies much more for group A, whereas group B performed consistently well. In fact, the level of completion of the ROS task and whether or not a participant filled in the questionnaire about the µRT task are highly correlated: *ρ* = 0.678. This finding suggests that many students had issues understanding the concepts of publish–subscribe and RPC interaction in general and could not apply those with either middleware. Although discrepancies between the two groups are not as pronounced for the time required and the number of requests for assistance, where average and median values are similar, variances of the answers to these questions are also much higher for group A.

Participants, on average, required 1/2 to one more hour to complete the line-following task with µRT than using ROS. Participants also asked for assistance five times more often when working on the µRT task. These two findings are again correlated with *ρ* = 0.746 for ROS and *ρ* = 0.809 for µRT. This indicates that participants could have completed the tasks in less time if they had been able to solve them completely on their own. While the overall higher number of requests for assistance for µRT shows that tutorial and documentation need to be improved, a participant stated for the µRT tutorial task that two of five requests for assistance were due to hardware issues with the robot rather than about µRT. This comment indicates that difficulties of understanding are not five times higher for µRT. However, as such differentiation was not considered in the study design, no definitive statement can be made based on the obtained data.

**FIGURE 10:**
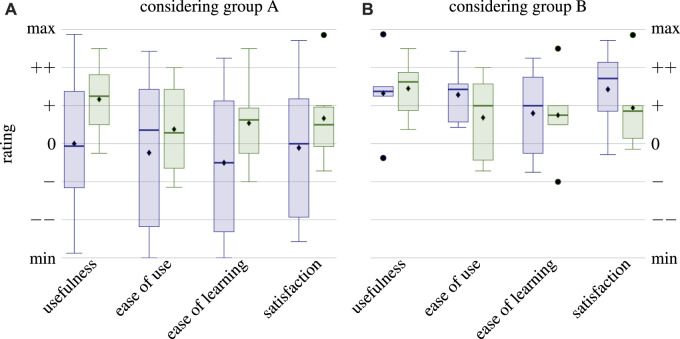
Statistical evaluation of the USE questionnaires. Each pair of box plots depicts results for ROS ((left)) () and µRT (right) (), respectively. In the axis on the left-hand side **(A)**, all participants are considered, although only six participants filled in the questionnaire for µRT while 12 participants did so for ROS. On the right-hand side **(B)**, only five participants who filled in both questionnaires are considered.

Results of the USE questionnaires are depicted in Figure 10 and show strong differences between groups A and B. Although µRT seems to outperform ROS in all four aspects when considering group A, results are more heterogeneous for group B. Due to the small size of the latter, the exact values depicted in Figure 10B cannot be considered significant. However, the overall positive trend for all aspects of USE and both middlewares is evident.

**FIGURE 11:**
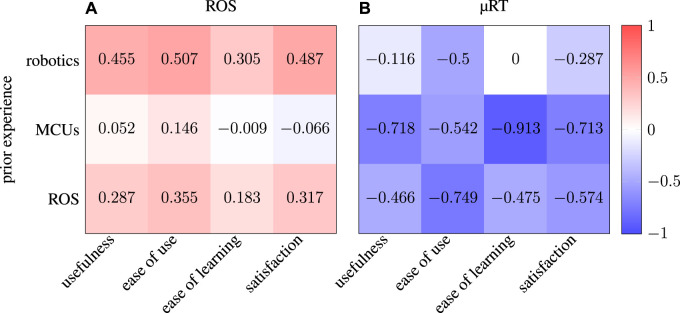
Correlation matrices showing the correlation between previous experience in three topics of computer science and the four aspects of the USE questionnaires. The left-hand side **(A)** depicts the correlation matrix regarding the USE questionnaire of the ROS line-following task (based on 12 data points), whereas the right-hand side **(B)** relates to µRT (based on six data points).

When investigating the correlations between previous experience in various areas of computer science and the information given in the USE questionnaires depicted in Figure 11, several further observations were made. First, the results of the USE questionnaire regarding the ROS task are correlated to prior experience in robotics but are uncorrelated to MCUs, both of which meet expectations. When investigating the correlation matrix regarding µRT, the first thing that stands out is the consistently inverse correlations. In particular, the strong (inverse) correlations with prior experience with MCUs and ROS are noteworthy but difficult to interpret. Either developers without previous knowledge in these topics rate µRT positively, or those who already have such experience rate µRT negatively, or both. Again, no clear conclusion can be drawn due to the small sample sizes (12 and 6 participants, respectively), so these findings should be considered and further investigated in future studies.

Finally, the USE questionnaires also contain qualitative questions, and some answers given by participants are worth mentioning. When asked about the most negative aspects of ROS and µRT, respectively, many complaints were made about the high complexity of ROS and the insufficient documentation of µRT. There were also some comments about µRT not supporting object-oriented programming languages such as C**++** and Python. On the contrary, many praised ROS that it supports exactly those languages and that it is an open-source project with a large community supporting it. However, positive mentions about µRT were its simplicity and that it is “not overloaded with features.”

## 4 Discussion

**FIGURE 12:**
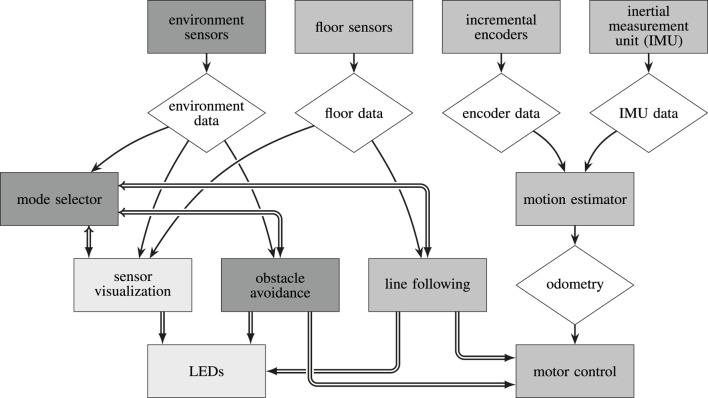
Example architecture as implemented on the AMiRo platform. Rectangles depict nodes, whereas topics are visualized as diamond shapes. Publish–subscribe interaction is indicated by 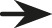. 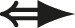
and 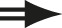
represent complete and “fire-and-forget” RPC interaction, respectively. Various shadings of nodes indicate that those are executed on different MCUs. Communication is hence tunneled over CAN by bridge nodes as required (not depicted).

As mentioned at the very beginning of this work (*cf.* section 1), µRT was developed as part of the software ecosystem of the AMiRo platform (Schöpping et al., 2015; Herbrechtsmeier et al., 2016). In addition to the evaluation results presented in section 3, practical experience was already gained by restructuring the entire real-time software of AMiRo to take advantage of the novel middleware and split the monolithic software into multiple applications (Schöpping and Kenneweg, 2022b). The new architecture allows arranging such applications among the several MCUs of AMiRo (*cf.* Herbrechtsmeier et al., 2016; Herbrechtsmeier, 2017) by means of *configurations*. Because all MCUs communicate *via* a common CAN interface (ISO, 1993), an according bridge node has been implemented and used to combine the individual µRT instances of all MCUs into a single virtual environment. An example configuration is depicted in Figure 12, which allows a user to select from four modes: idle (no action executed at all), sensor visualization (sensor readings are visualized *via* LEDs), line following (AMiRo follows a line on the ground), and obstacle avoidance (AMiRo moves forward. However, it avoids any encountered obstacles). Although the motor control loop is entirely processed on the same MCU (*cf.* right part of Figure 12), control logic, sensor input, and visualization are distributed among the entire robot. In order to change the behavior of AMiRo, many nodes can be reused, whereas others are modified or replaced, or even further nodes are added to the system.^12^ All in all, µRT already proved its benefits and effectiveness as it is employed as a standard tool for the productive use of AMiRo in educational and scientific contexts.

### 4.1 Conclusion

In this work, a novel, real-time, capable middleware—µRT—was presented. In contrast to existing solutions, it is used in resource-constrained platforms, such as microcontrollers (MCUs), and features validation of timing constraints at runtime. µRT provides two communication schemes, publish–subscribe and future RPCs, and overall offers a similar feature set to popular middlewares such as ROS 2. This work also presented a thorough evaluation of µRT, including a feature comparison with existing solutions, an in-depth performance analysis, and a usability study, which assessed the experience software developers had with µRT. Although the middleware showed excellent results overall, some issues and potential areas for improvement were identified. Findings of all analyses regarding µRT are summarized by recalling the initial goals as defined at the very beginning of section 2.

1. While the memory footprint of µRT is slightly higher than that for existing solutions targeting MCUs, it is still reasonably small.
2. Real-time capabilities were suitable for even hard real-time use cases with strict timing constraints.
3. Performance of µRT scales linearly in most regards, but some aspects concerning hard real-time interaction exhibit slightly exponential scaling.
4. µRT allows validating real-time constraints at runtime, a unique feature among all middlewares to our knowledge.
5. Topic-based publish–subscribe interaction is provided for periodic/time-based communication, and RPCs allow for event-based interaction.
6. While the usability study proved the effectiveness of µRT for developers, current API documentation is lacking.
7. µRT can interact with other middlewares by means of dedicated bridge nodes.
8. Depending on the requirements of individual use cases, the feature set and performance characteristics of µRT can be optimized by its fine-grained configurability.

### 4.2 Future prospect

The most pressing drawback of µRT is its lacking documentation. Although a tutorial exists and HTML-based documentation of the entire API is provided, the usability study revealed that both are insufficient in their current state (*cf.* section 3.3.2). Another complaint of participants in the study was the missing support of popular object-oriented programming languages, such as C**++** and Python. This demand was already considered with the design of µRT, so that the according wrappers of its API and integration in further tools, such as GenoM (Mallet et al., 2010) or BRICS (Bruyninckx et al., 2013), can be realized with relative ease. This study also revealed several aspects which should be investigated further, as no clear conclusions could be drawn from the data obtained so far because of the study design and sample size. Another lacking feature of µRT, or more precisely the ecosystem on top, is bridge nodes interfacing other popular middlewares such as ROS 2 or MQTT (*cf.* section 2.8). Although those are not part of µRT itself, the availability of such interface nodes would most probably benefit its adoption for other platforms. Due to its focus on real-time capability and validation of timing constraints at runtime, some compromises were made with the design of µRT. First, its current implementation makes extensive use of mutex locks but does not feature preferable lock-free methods. Whether such are actually possible and what benefits this would bring for µRT need further investigation. Moreover, performance analyses showed nonlinear scaling in some situations (*cf.* section 3.2.2). As this can become an issue for large, highly complex systems, performance in this regard needs to be further improved. Finally, some proposed enhancements include providing a fallback event system if no external implementation is mapped to its operating system abstraction layer (OSAL; *cf.* section 2.4).

As µRT has already matured to the point that it has become a core component of the AMiRo software habitat, many of these issues and requested features will be addressed in the future. By the regular employment of the platform for teaching, user feedback on changes made to the system can be obtained quickly, and µRT will be refined in the upcoming years. Assuming that µRT will be accepted by the robotics community and adopted to further platforms, development could be accelerated thanks to its open-source approach.

## Data Availability

The datasets presented in this study can be found in online repositories. The names of the repository/repositories and accession number(s) can be found at: https://gitlab.ub.uni-bielefeld.de/uRT-evaluation-data/ROS-2-pendulum-demo https://gitlab.ub.uni-bielefeld.de/uRT-evaluation-data/uRT-performance-benchmark https://gitlab.ub.uni-bielefeld.de/uRT-evaluation-data/uRT-usability-study.

## Footnotes

1 CPU: Intel Core i7-7567U (2 cores) locked at 3.5 GHz and hyper-threading disabled; OS: Ubuntu 22.04 with real-time kernel (Linux 5.15.0–1016-realtime x86_64); ROS: “Humble Hawksbill”

2
https://gitlab.ub.uni-bielefeld.de/AMiRo/uRtWare; licensed under LGPL 3

3 Modification of *t*_info,*i*_ is possible if desired

4 While timestamps are defined by µRT to be 64-bit wide, delays may be represented by a smaller type

5 Although it is recommended to synchronize *nodes*, the implementation actually works on a thread level

6 Retrieval of a *topic* by its identifier has linear runtime complexity: O(#topics)

7 Retrieval of a *service* by its identifier has linear runtime complexity: O(#services)

8 In fact, actions in ROS 2 are composed of multiple topics and services as well

9 Due to the wide support of C by most programming languages, additional interface layers can be added to ease development (*cf.* section 4.2)

10 STM32F1 (Cortex-M3), STM32F4 (Cortex-M4), STM32F7 (Cortex-M7), STM32-G0 (Cortex-M0+), and STM32-L4 (Cortex-M4)

11 Software environment: AMiRo-OS version 2.3 (Schöpping et al., 2016), which incorporates ChibiOS version 21.11 (di Sirio, 2022); *cf.* Schöpping and Kenneweg, 2022b, commit e8ddcd253bb5996509cb82adde700b0fec7798f1

12 Actually, several more applications are already implemented for AMiRo (e.g., for power management), which are not depicted in Figure 12.
